# The Association of Alternate VEGF Ligands with Resistance to Anti-VEGF Therapy in Metastatic Colorectal Cancer

**DOI:** 10.1371/journal.pone.0077117

**Published:** 2013-10-15

**Authors:** Christopher H. Lieu, Hai Tran, Zhi-Qin Jiang, Muling Mao, Michael J. Overman, E. Lin, Cathy Eng, Jeffrey Morris, Lee Ellis, John V. Heymach, Scott Kopetz

**Affiliations:** 1 Division of Medical Oncology, University of Colorado, Denver, Colorado, United States of America; 2 Department of Gastrointestinal Medical Oncology, the University of Texas MD Anderson Cancer Center, Houston, Texas, United States of America; 3 Department of Thoracic/Head & Neck Medical Oncology, the University of Texas MD Anderson Cancer Center, Houston, Texas, United States of America; 4 Department of Biostatistics, the University of Texas MD Anderson Cancer Center, Houston, Texas, United States of America; 5 Department of Surgical Oncology, the University of Texas MD Anderson Cancer Center, Houston, Texas, United States of America; UT MD Anderson Cancer Center, United States of America

## Abstract

**Background:**

Circulating angiogenic factors are altered in patients with mCRC receiving bevacizumab. Evaluation of alterations in levels of VEGF ligands may provide insights into possible resistance mechanisms.

**Methods:**

PlGF, VEGF-A, VEGF-C, and VEGF-D were measured from two cohorts of patients. Sequential plasma samples were obtained from a discovery cohort of 42 patients treated with chemotherapy and bevacizumab. A validation cohort included plasma samples from a cross-sectional of 403 patients prior to chemotherapy, or after progression on a regimen with or without bevacizumab.

**Results:**

In the discovery cohort, VEGF-C was increased prior to progression and at progression (+49% and +95%, respectively, p<0.01), consistent with previously reported elevations in PlGF. Levels of VEGF-D were increased (+23%) at progression (p=0.05). In the validation cohort, samples obtained from patients after progression on a regimen with bevacizumab had higher levels of PlGF and VEGF-D (+43% and +6%, p=0.02, p=0.01, respectively) compared to untreated patients, but failed to validate the increase in VEGF-C seen in the first cohort. Patients who progressed on chemotherapy with bevacizumab had significantly elevated levels of PlGF (+88%) but not VEGF-C and VEGF-D compared to patients treated with chemotherapy alone. Elevations of PlGF and VEGF-D appeared transient and returned to baseline with a half-life of 6 weeks.

**Conclusions:**

Increases in PlGF and VEGF-D were observed after progression on chemotherapy with bevacizumab. These changes appear to be reversible after discontinuing therapy. These ligands are associated with resistance to bevacizumab-containing chemotherapy in mCRC, but causation remains to be established.

## Introduction

Angiogenesis is an essential process for both tumor growth and metastatic spread of disease [1,2]. During tumorigenesis, the balance of proangiogenic factors, growth factors, and cytokines that regulate angiogenesis is disrupted and the “angiogenic switch” is increasingly recognized as a rate-limiting secondary event in multistage carcinogenesis [2–5]. Vascular endothelial growth factor-A (VEGF-A) is a key growth factor for endothelial cells in patients with CRC [6,7]. The addition of the monoclonal antibody, bevacizumab, has improved the overall survival of patients with mCRC [8,9]. 

Despite the clinical benefit provided by bevacizumab, it is also well-recognized that many patients have re-established angiogenesis despite continued VEGF-A blockade [10]. Preclinical work has suggested that alternate proangiogenic factors may modulate sensitivity to anti-VEGF-A therapy and allow regrowth of tumor-associated vasculature [11–13]. Members of the VEGF signaling family aside from VEGF-A have been implicated in angiogenesis, including placental growth factor (PlGF), VEGF-C, and VEGF-D [14,15]. However, the role of alternate VEGF ligands in angiogenesis remains controversial. Some data in the literature report that PlGF enhances pathological angiogenesis by initiating a cross-talk between VEGFR-1 and VEGFR-2, but other studies have failed to confirm these findings [16–19]. VEGF-C has been associated with angiogenesis in breast cancer and has been shown to synergize with basic fibroblast growth factor and VEGF-A to induce angiogenesis, but another study has suggested that VEGF-C induces blood vessel changes without evidence of new angiogenesis [20–22]. There is less data on the role of VEGF-D and angiogenesis, but a study of patients with CRC found that lower expression of VEGF-D was associated with greater benefit from treatment with bevacizumab [23]. 

Cytokine and angiogenic factors (CAFs) are modulated in patients with mCRC after receiving bevacizumab-containing chemotherapy [24,25]. In one study of bevacizumab in rectal cancer, bevacizumab monotherapy significantly increased plasma PlGF as well as free VEGF-A [25]. Cytokine analysis in a single-arm phase II study with FOLFIRI and bevacizumab demonstrated that alternate proangiogenic cytokines are modulated by chemotherapy and bevacizumab and increases were seen before disease progression in a subset of patients [24]. However, this study did not fully evaluate alternate VEGF ligands nor allow separation of the separate effects of the cytotoxic chemotherapy and bevacizumab. The primary objective of this study, therefore, is to determine alterations in the alternate VEGF ligands, including PlGF, VEGF-C and VEGF-D, in patients receiving bevacizumab-containing chemotherapy. 

## Methods

**Figure 1:**
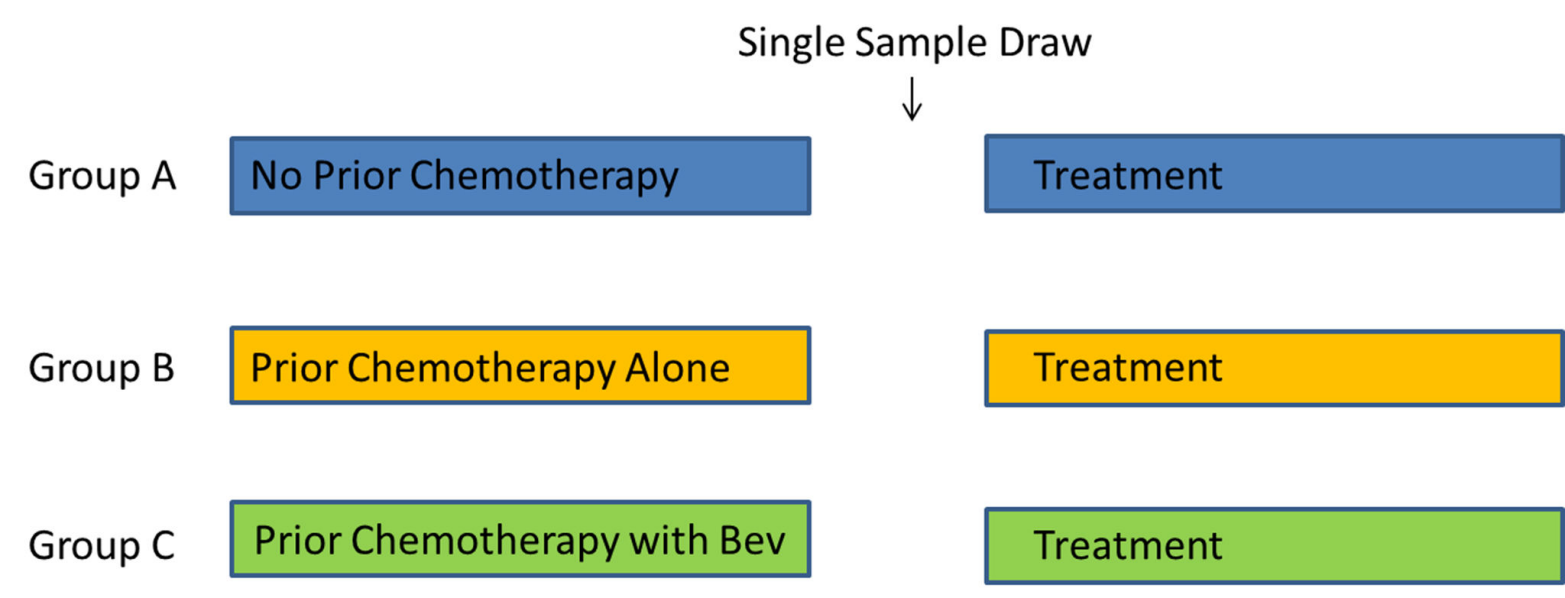
Study Schema.

All research involving human participants was approved by the Institutional Review Board of the University of Texas MD Anderson Cancer Center. All participants provided their written informed consent to participate in this study. The ethics committee of the University of Texas MD Anderson Cancer Center approved this consent procedure. Two cohorts were evaluated during this study (Figure 1). The discovery cohort was developed from plasma acquired from 42 patients with mCRC treated on a phase II clinical trial with FOLFIRI and bevacizumab [24]. Venous blood was drawn into EDTA tubes and immediately processed for plasma at baseline, before each cycle of chemotherapy (including a sample 2 weeks after single-agent bevacizumab in the first cycle), after first restaging, and at the time of progression. Plasma collection included serial centrifugation to deplete platelets, which have otherwise been shown to affect free cytokine levels. On retrospective review of computed tomography imaging, we identified the plasma sample associated with the best radiographic response for all patients. This sample (henceforth denoted as “before progression”) represented a point before the development of progressive disease.

The discovery cohort was derived from plasma obtained from 42 patients with mCRC treated on a single-arm phase II study with FOLFIRI and bevacizumab. Plasma was obtained at baseline, immediately before each cycle of chemotherapy, and at the time of progression. The validation cohort utilized a cross-section of patients with mCRC who underwent a one-time plasma collection between 2002 and 2008. 

The validation cohort utilized a cross-section of patients with mCRC who underwent a one-time plasma collection between 2002 and 2008. A single blood sample from 784 patients with mCRC was obtained upon first visit to our center. The blood was collected in EDTA tubes and underwent centrifugation at 2800rpm for 10 minutes at -22°C followed by aliquoting of the plasma into 0.5mL cryovials and storage at -80°C. Samples were collected with repeated centrifugation to deplete platelets prior to freezing. All treatment histories were reviewed for prior treatment regimens, prior treatment duration, time from last chemotherapy or bevacizumab to plasma sample collection, and number of metastatic disease sites. Patients were separated into 3 groups based on the treatment history at the time of plasma sampling: patients prior to receiving any treatment for their cancer (Group A), patients progressing on chemotherapy without bevacizumab (Group B), and patients progressing on a chemotherapy regimen that included bevacizumab (Group C). 

Matched pairs of samples were identified to allow comparison of Groups A and C, and separate pairs identified to compare Groups B and C. In order to control for disease burden and prior therapy, patient samples were matched for number of metastatic disease sites (Groups A, B, C), prior chemotherapy duration, and time from last chemotherapy dose to sample collection (Groups B, C). Matching was done in R using the Optmatch routine, resulting in 169 pairs for Groups A and C, and 65 pairs for Groups B and C [26]. 

Plasma samples were thawed in parallel and assayed in duplicate. Samples were placed randomly onto plates, keeping paired samples on the same plate. For each individual sample, the coefficient of variance (CV%) was calculated, with a repeat analysis if the CV% was greater than 25%. Levels of cytokines, including VEGF-A and PlGF were measured by multiplex bead assay (BioRad Pro Human Cytokine 23- and 27-plex Assay). Levels of VEGF-C and VEGF-D were measured by enzyme-linked immunosorbent assays (R&D Systems Human VEGF-C Immunoassay Catalog #DVEC00, R&D Systems Human VEGF-D Immunoassay Catalog #DVED00) and analyzed in duplicate with no more than one prior freeze-thaw cycle [27].

Comparisons were performed using the two-sided, nonparametric Wilcoxon paired test, with p<0.05 significance. Spearman rank correlation coefficients were computed to assess the correlation between the time from last chemotherapy dose to sample collection and specific cytokine levels. Data were plotted using vioplot package in R [28]. Data was fit to a single-phase log decay to identify half-life of the elevations. Correlation among cytokine levels were assessed by Pearson correlation coefficients computed on the log-transformed cytokine values. To evaluate whether VEGF-A, VEGF-C, VEGF-D, or PlGF were prognostic, evaluable patients in the no prior therapy group within the validation cohort were split into two cohorts based on their median cytokine levels and analyzed using univariate analysis (Kaplan-Meier) and multivariate analysis (Cox-regression). All computations were carried out using SAS (SAS institute, Cary, NC). 

## Results

Paired plasma samples were obtained for 42 patients in the discovery cohort, and 403 patients in the validation cohort. Patient characteristics are shown in Table 1. For the validation cohort, samples were obtained a median of 3 weeks after treatment with a regimen with chemotherapy alone or chemotherapy with bevacizumab. Samples were well matched for the pre-specified variables. 

**Table 1:** Characteristics of patients included in the study.

	**Discovery cohort**	**Validation Cohort**				
		**No chemotherapy (Group A)**	**Chemotherapy w/ Bev (Group C)**		**Chemotherapy w/o Bev (Group B)**	**Chemotherapy w/Bev (Group C)**
Number of samples	179	169	169		65	65
Number of Patients	42	169	169		65	65
Age, years (median)	57	58	53		53	53
Male (%)	43%	58%	62%		57%	62%
Primary Disease Site (%)						
Rectum	14%	13%	15%		23%	20%
Colon	86%	87%	85%		77%	80%
Adenocarcinoma Histology (%)						
Moderately Differentiated	86	88	98		95	100
Poorly Differentiated	14	12	2		5	0
Number of metastatic sites (avg)	1.7	1.7	1.7		1.4	1.4
Duration of prior chemotherapy (months)	N/A	N/A	6		5	6
Prior Chemotherapy Received						
5-Fluorouracil/Capecitabine (%)	0	0	100		100	100
Oxaliplatin-Based (%)	0	0	89		40	88
Irinotecan-Based (%)	0	0	58		43	45
Bevacizumab (%)	0	0	100		0	100
EGFR-inhibitor (%)	0	0	26		6	15
Last chemotherapy to sample collection (days)	N/A	N/A	N/A		35	29
Average LDH	1372	1129	821		616	821
Median Platelet Count	N/A	333	222		238	229

### VEGF-D, and PlGF Are Increased at the Time of Progression of Chemotherapy with Bevacizumab

**Figure 2:**
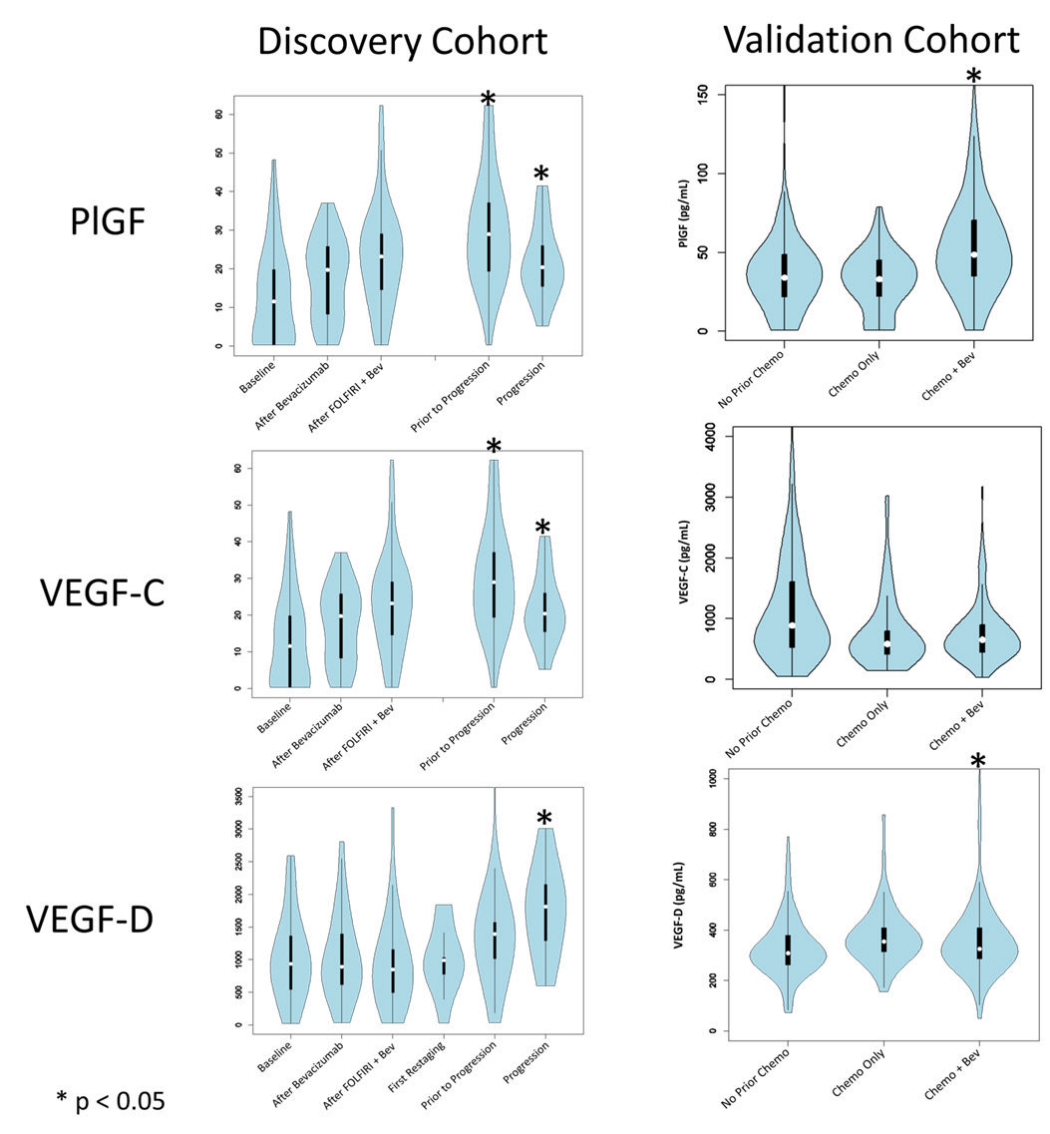
Levels of alternate VEGF ligands in cohort 1 and cohort 2. Error bars represent standard error of the mean. Following treatment with FOLFIRI and bevacizumab in the discovery cohort, VEGF-C was increased prior to progression and at the time of progression. These findings were similar to previously reported changes from this cohort in PlGF. Levels of VEGF-D were increased at the time of progression, but in contrast to VEGF-C, levels of VEGF-D were not increased prior to progression. In the validation cohort, PlGF was elevated in patients previously receiving chemotherapy and bevacizumab. VEGF-D was also minimally elevated in patients previously receiving chemotherapy and bevacizumab, but elevations in VEGF-C seen in the discovery cohort were not replicated in the validation cohort.

Following treatment with FOLFIRI + bevacizumab in the discovery cohort, VEGF-C was increased prior to progression and at the time of progression (+49% from baseline [p=0.045] and +94% [p=0.004], respectively) (Table 2 and Figure 2). These findings were similar to previously reported changes from this cohort in PlGF [24]. Levels of VEGF-D were increased at the time of progression (+23%, p=0.04), but in contrast to VEGF-C, levels of VEGF-D were not increased prior to progression (-1%, p=0.84).

**Table 2:** Alternate VEGF Ligand Cytokine Levels.

**DISCOVERY COHORT**																	
	**Baseline**		**Post-Bevacizumab**			**Post-FOLFIRI+B**			**1st Restaging**			**Prior to Progression**			**Progression**		
**Parameter**	**mean (pg/mL)**	**median (pg/mL)**	**mean (pg/mL)**	**median (pg/mL)**	**% baseline**	**mean (pg/mL)**	**median (pg/mL)**	**% baseline**	**mean (pg/mL)**	**median (pg/mL)**	**% baseline**	**mean (pg/mL)**	**median (pg/mL)**	**% baseline**	**mean (pg/mL)**	**median (pg/mL)**	**% baseline**
PlGF	12.6	12.4	17.4	20.6	166%	23.0	23.2	187%	N/A	N/A	N/A	29.9	29	234%	21.6	20.4	165%
VEGF-C	1020.3	936.6	1026.6	891.5	95%	947.3	850.0	91%	1002.7	991.7	106%	1461.9	1393.3	149%	1748.9	1813.3	194%
VEGF-D	238.2	240.5	244.5	243.1	101%	247.7	238.5	99%	272	304.1	126%	232.7	249.1	104%	331.5	295	123%

In order to validate findings in the discovery cohort, we compared changes in VEGF ligands from untreated patients and matched patients previously treated with chemotherapy and bevacizumab (Table 3). Consistent with the discovery cohort, the chemotherapy with bevacizumab group had higher levels of PlGF (+32%, p<0.0001). Likewise, levels of VEGF-D were elevated in patients previously treated with chemotherapy and bevacizumab, although the magnitude of increase was lower than that seen in the first cohort (+6%, p=0.018). The elevation in VEGF-C levels seen in the discovery cohort was not replicated in the validation cohort, which instead demonstrated lower levels of VEGF-C (-36%, p<0.0001) in patients previously treated with chemotherapy and bevacizumab. 

**Table 3:** Validation Cohort.

**VALIDATION COHORT**															
**No Chemotherapy (Group A)**					**Chemotherapy with Bev (Group C)**				**Chemotherapy without Bev (Group B)**			**Chemotherapy with Bev (Group C)**			
**Parameter**	**mean (pg/mL)**	**median (pg/mL)**	**% compared to Group C**	**mean (pg/mL)**		**median (pg/mL)**	**% compared to Group A**	**mean (pg/mL)**		**median (pg/mL)**	**% compared to Group C**		**mean (pg/mL)**	**median (pg/mL)**	**% compared to Group B**
PlGF	36.9	34	70%	54.2		48.7	143%	31.9		33.1	53.13%		63.7	62.3	188.22%
VEGF-C	1138	889.6	136%	751.4		652.7	73%	770.3		586	95.55%		737.6	613.3	104.66%
VEGF-D	326.5	308.5	95%	363.6		325.7	106%	374.6		354.1	93.31%		404.8	379.5	107.17%

We also evaluated levels of VEGF-A in untreated patients and patients receiving chemotherapy without bevacizumab. In the untreated group, the median level of VEGF-A was 760pg/mL (SD 1474pg/mL), and in the chemotherapy without bevacizumab group, the median level of VEGF-A was 534pg/mL (SD 599pg/mL). In the chemotherapy with bevacizumab group, VEGF-A levels were significantly higher with a median of 1740pg/mL (SD 3702pg/mL), but interpretation of the levels of VEGF-A in the chemotherapy with bevacizumab group are confounded due to the presence of bevacizumab-bound VEGF-A. 

### Increased PlGF Is Attributed to Bevacizumab Treatment

Because the design of the discovery cohort could not distinguish the separate impact of chemotherapy and bevacizumab, we compared patients who had received chemotherapy alone with patients who had received prior bevacizumab and chemotherapy. Compared to the chemotherapy without bevacizumab group, the chemotherapy with bevacizumab group had significantly elevated levels of PlGF (+72%, p<0.0001). However, there was no difference in VEGF-C (+5%, p=0.64) and VEGF-D (+7%, p=0.18) in patients receiving chemotherapy alone and patients receiving prior chemotherapy and bevacizumab. If this finding is confirmed, it would suggest that any changes in VEGF-C and VEGF-D may be due to the impact of the disease progression and prior chemotherapy and not a direct result of bevacizumab. 

### VEGF-A is correlated with progression-free survival and overall survival

**Figure 3:**
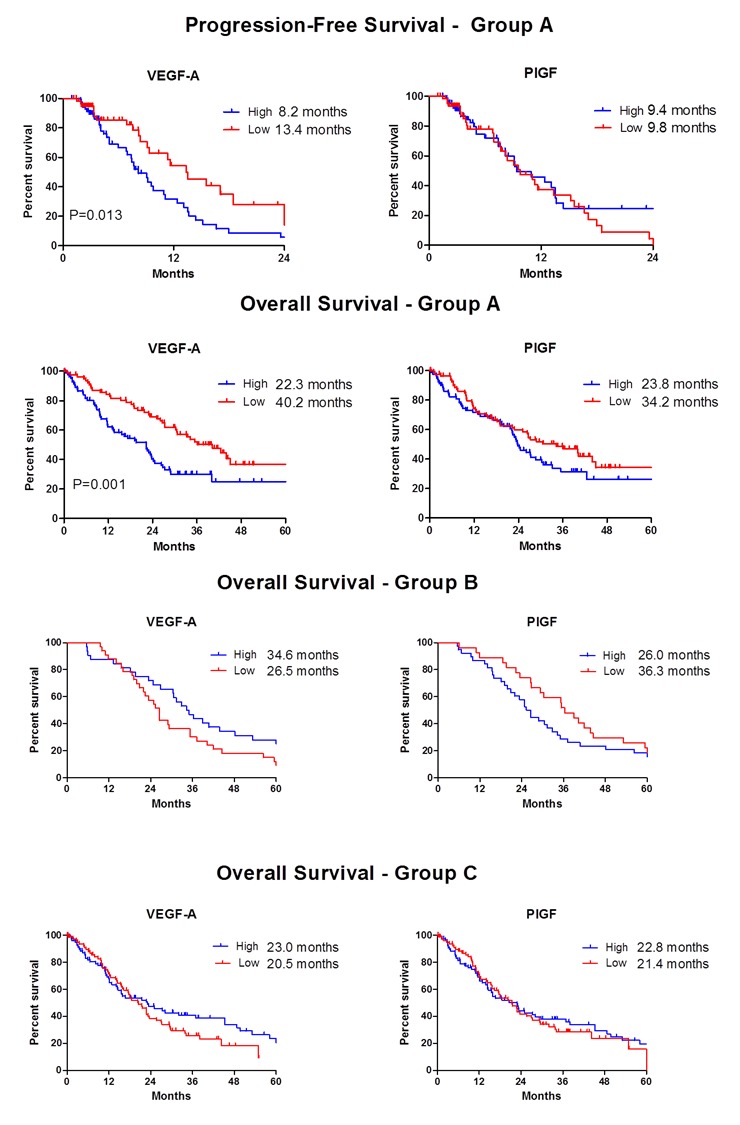
Progression-free survival and Overall Survival in high and low VEGF-A and PlGF. To evaluate whether VEGF-A, VEGF-C, VEGF-D, or PlGF were prognostic, evaluable patients in the untreated group were split into two cohorts based on their median cytokine levels. VEGF-C, VEGF-D, and PlGF were not correlated with clinical outcomes. However, VEGF-A was associated with longer progression-free and overall survival. There was no correlation between the level of any VEGF ligand and the subsequent clinical outcomes in patients previously treated with or without bevacizumab.

To evaluate whether VEGF-A, VEGF-C, VEGF-D, or PlGF were prognostic, evaluable patients in the no prior therapy group within the validation cohort were split into two cohorts based on their median cytokine levels. Baseline levels of VEGF-C, VEGF-D, and PlGF were not correlated with clinical outcomes. However, on univariate analysis low VEGF-A was associated with a longer progression-free (p=0.001) and overall survival (p=0.013), as has been previously demonstrated (Figure 3) [29]. This correlation was not statistically significant on multivariate analysis. There was no correlation between the level of any VEGF ligand and subsequent clinical outcomes in patients previously treated with or without bevacizumab (Figure 3).

### Elevations in PlGF and VEGF-D after chemotherapy are transient

**Figure 4:**
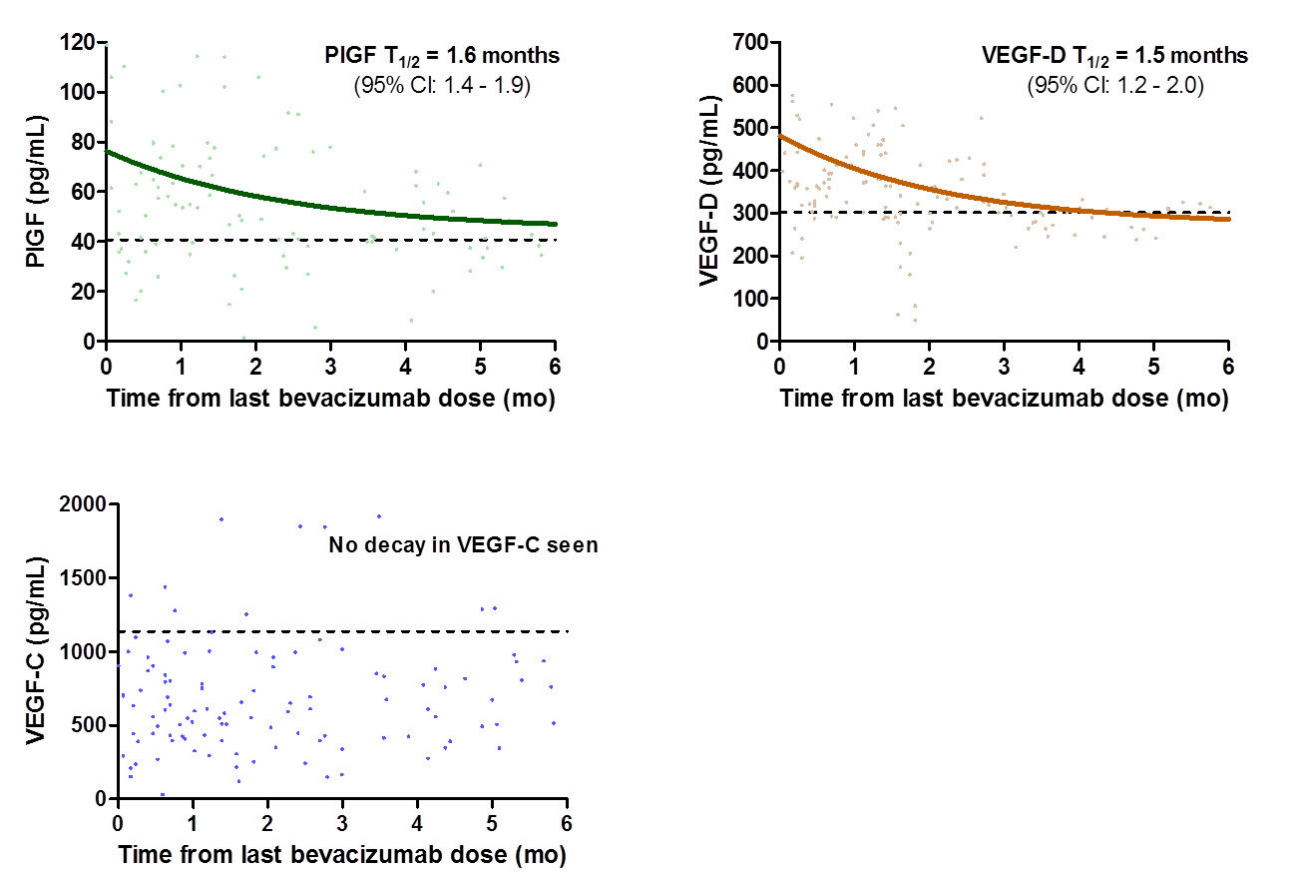
Levels of alternate VEGF ligands decrease following discontinuation of bevacizumab. The Spearman correlation method was applied to determine the correlation between the time to last chemotherapy dose to sample collection and specific cytokine levels. Levels of PlGF and VEGF-D were negatively correlated with the time from last bevacizumab dose to sample collection (p<0.0001) suggesting that elevations in these cytokines were transient and not maintained after removal of bevacizumab containing chemotherapy. No decay in VEGF-C was observed.

The Spearman correlation method was applied to determine the correlation between the time of final chemotherapy administration to sample collection and specific cytokine levels in the validation cohort. Levels of PlGF and VEGF-D negatively correlated with the time from last bevacizumab administration to plasma collection (p<0.0001) suggesting that elevations in these cytokines were transient and not maintained after removal of bevacizumab containing chemotherapy (Figure 4). No such correlation was seen in patients treated with chemotherapy alone. In order to determine the half-life of the elevations, the data was fit to a single-phase log decay curve. The half-life for PlGF was found to be 1.6 months (95%CI 1.4-1.9), and the half-life for VEGF-D was found to be 1.5 months (95%CI 1.2-2.0).

### Correlation of VEGF ligands with alternate cytokines

**Figure 5:**
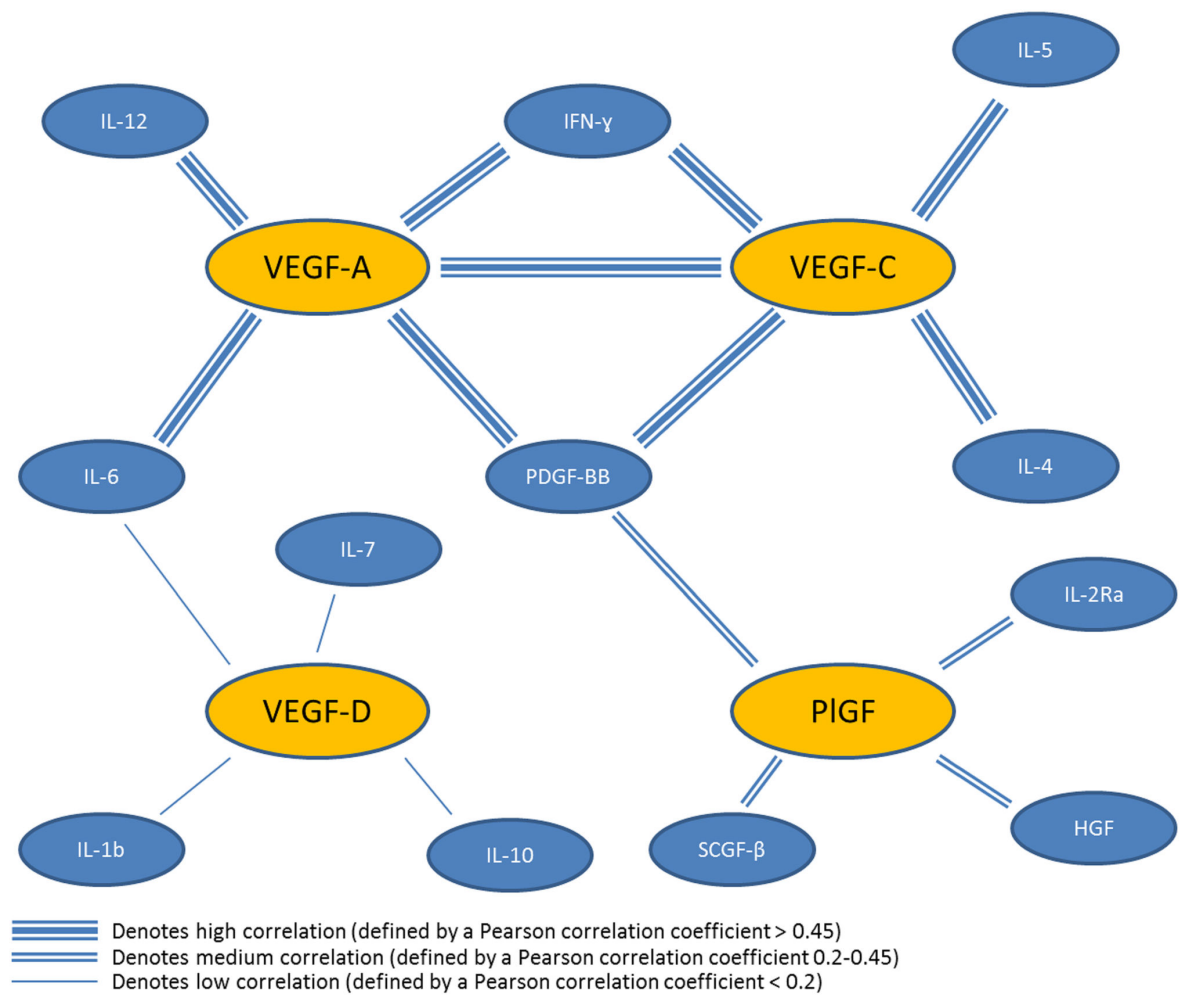
Correlation of alternate VEGF ligands with alternate angiogenic and inflammatory cytokines. Several cytokines were highly correlated with VEGF-A, VEGF-C, and PlGF. VEGF-D showed very little correlation with other cytokines. PDGF-BB was highly correlated with VEGF-A, VEGF-C, and PlGF and IFN-ɣ correlated with both VEGF-A and VEGF-C. VEGF-A and VEGF-C were also highly correlated with each other, but there was no significant correlation between the other VEGF ligands.

Prior studies have not evaluated the correlation of VEGF family members with each other or alternate angiogenic and inflammatory cytokines. To explore these relationships, we utilized the second untreated patient cohort to evaluate cytokines that were correlated with one or more of the VEGF ligand family members. We found that several cytokines were highly correlated with VEGF-A, VEGF-C, and PlGF (Figure 5). Interestingly, VEGF-D showed very little correlation with other cytokines. PDGF-BB was highly correlated with VEGF-A, VEGF-C, and PlGF and IFN-ɣ correlated with both VEGF-A and VEGF-C. VEGF-A and VEGF-C were also highly correlated with each other, but there was no significant correlation between the other VEGF ligands.

## Discussion

This study represents a large evaluation of alternate VEGF ligands after progression on anti-VEGF therapy and chemotherapy in a prospective cohort and retrospective cohort of patients with mCRC. The analysis of these specific cytokines demonstrates that alternate VEGF ligands are modulated by bevacizumab and chemotherapy. PlGF has previously been shown to be elevated prior to progression and at the time of progression on a bevacizumab regimen [24,25]. In a larger retrospective cohort, these elevations in PlGF were seen after chemotherapy and bevacizumab but not after chemotherapy alone, suggesting that changes seen in PlGF are specific to those patients receiving bevacizumab. PlGF levels in plasma and tumors correlate with tumor stage, vascularity, recurrence, metastasis, and survival in various tumors [30,31] and has also been shown to increase cell sensitivity to VEGF-A [32]. Results from a randomized Phase III trial of ziv-aflibercept (a dual-inhibitor of PlGF and VEGF-A) in patients that have progressed on prior chemotherapy demonstrate that continued blockade of VEGF family members is beneficial for patients with acquired resistance to bevacizumab, although further studies will be required to separate the relative contributions of PlGF and VEGF-A inhibition. In human tumor cell lines inhibited by anti-PlGF antibodies, one study found that the efficacy of anti-PlGF strongly correlated with VEGFR-1 expression in tumor cells but not with antiangiogenesis [33]. 

Levels of VEGF-C were elevated in the prospective cohort prior to progression and at the time of progression in patients progressing on a bevacizumab regimen. However, this elevation was unable to be confirmed in our larger retrospective cohort. Furthermore, there was no difference seen between patients receiving chemotherapy alone and patients receiving chemotherapy and bevacizumab. It is unclear why the elevation in VEGF-C was unable to be confirmed. Repeated freeze-thaw and extended room temperature exposure did not substantially alter VEGF-C levels. Although platelets were depleted by centrifugation, there may be potential for some leakage of these cytokines from platelet reservoirs during processing. The heterogeneous patient population represents a possible limitation in the validation cohort. It is also possible that the elevations seen in VEGF-C in the prospective cohort may consist in more transient elevations that aren’t detectable at a mean of 4 weeks post-bevacizumab in the second cohort. Given that the half-life of bevacizumab is 20 days, this may have also influenced the results seen for VEGF-C.

Modest elevations were seen in VEGF-D in patients progressing on a bevacizumab regimen. This elevation was confirmed in the retrospective cohort, but the magnitude of the elevation is of unclear biological significance. There was no statistically significant difference between patients receiving chemotherapy only and patients receiving chemotherapy with bevacizumab, although the magnitude of the increase was similar. It should also be noted that levels of VEGF-D were very tightly regulated with low inter-patient variability. In contrast, levels of VEGF-C had greater variation within the population, with approximately 4 fold higher standard deviation. This may suggest that slight variations in VEGF-D may lead to larger biologic effects, but further study will need to be performed in order to evaluate the biologic effect of changes in VEGF-D.

Within the retrospective cohort, we found that levels of PlGF and VEGF-D were negatively correlated with the time from last bevacizumab dose to sample collection. This suggests that changes in PlGF and VEGF-D at the time of radiographic progression are temporary following discontinuation of bevacizumab. Importantly, there was no temporal change in PlGF and VEGF-D in patients treated with chemotherapy only. These findings have a clear implication regarding the timing of therapeutic agents that target alternate VEGF ligands. Treating patients with agents directed against alternate VEGF ligands at the time of bevacizumab resistance or prior to radiographic progression may yield the greatest therapeutic efficacy as the levels of alternate VEGF ligands would be highest at that time. While the cytokine responses to bevacizumab are transient, it is unclear if this response represents a permanent renewing of angiogenesis that can be exploited with novel therapies. Future clinical studies may attempt to investigate the timing of the addition of these agents in coordination with plasma cytokine levels.

Prior studies have not evaluated the correlation of VEGF family members with each other or alternate angiogenic and inflammatory cytokines. Our results suggest that VEGF-A, VEGF-C, VEGF-D, and PlGF are not part of the same angiogenic response and are instead correlated with levels of alternate angiogenic cytokines. Elevations in alternate VEGF ligands are not evidence that these ligands cause restoration of angiogenesis and clinical resistance. Similarly, our data also raise the hypothesis that the elevations in alternate VEGF ligands appear to be associated with a larger collection of circulating angiogenic factors that may be collectively involved in re-establishing angiogenesis. These associations are important to delineate in future studies, as the complexities of angiogenesis suggest that many of these cytokines and chemokines work in concert to produce a microenvironment supportive for neovascularization. Oral tyrosine kinases have been developed with broad activity against a variety of tyrosine kinases, including regorafenib which has recently shown survival benefit in refractory CRC patients [34]. 

This study has several limitations. Because plasma was taken from patients at only one time point in the validation cohort, serial measurements of plasma CAFs were unable to be studied. The length of prior treatment was also heterogeneous in both groups B and C, though samples were matched for treatment duration as well as time from last chemotherapy dose to sample collection to account for this discrepancy. It is possible that varying schedules of bevacizumab were used in the validation cohort which may differ than the strict protocol-based regimen of bevacizumab in the discovery cohort. It is also unclear how well circulating levels of VEGF ligands actually reflect the tumor microenvironment. There is evidence to suggest that antiangiogenic therapies may elicit a host response rather than a malignant cell response that contributes to therapeutic resistance [35]. It is also difficult to determine what magnitude of change in cytokine levels would be necessary to evoke a biologic response. VEGF receptors have also been shown to play an important role in tumors treated with anti-VEGF therapy, as a recent study has shown that a locus in VEGFR1 correlates with increased VEGFR1 expression and poor outcome with bevacizumab treatment [36]. Finally, this study shows an association of alternate ligands with bevacizumab resistance, but is unable to show a true causative resistance mechanism. Further study will be needed in either preclinical models or in the setting of a clinical trial to answer these critical questions.

VEGF ligands other than VEGF itself are associated with bevacizumab-containing chemotherapy resistance in metastatic CRC. Further preclinical work may help elucidate which angiogenic factors can be targeted in order to suppress tumor angiogenesis. Further study will also be required to determine if these changes are causative for antiangiogenic resistance. With the recent availability of two new agents targeting VEGF signaling, translational studies will be critical to identify the best setting to utilize anti-angiogenic therapies.
